# Direct electrical stimulation enhances osteogenesis by inducing Bmp2 and Spp1 expressions from macrophages and preosteoblasts

**DOI:** 10.1002/bit.27142

**Published:** 2019-09-23

**Authors:** Kasama Srirussamee, Sahba Mobini, Nigel J. Cassidy, Sarah H. Cartmell

**Affiliations:** ^1^ Department of Materials The University of Manchester Manchester UK; ^2^ Instituto de Micro y Nanotecnología IMN‐CNM The Spanish National Research Council (CSIC) Madrid Spain; ^3^ Departamento de Biología Molecular and Centro de Biología Molecular “Severo Ochoa” (UAM‐CSIC) Universidad Autónoma de Madrid Madrid Spain; ^4^ Department of Civil Engineering University of Birmingham Birmingham UK

**Keywords:** bone regeneration, electrical stimulation, faradic by‐products, macrophages, preosteoblasts

## Abstract

The capability of electrical stimulation (ES) in promoting bone regeneration has already been addressed in clinical studies. However, its mechanism is still being investigated and discussed. This study aims to investigate the responses of macrophages (J774A.1) and preosteoblasts (MC3T3‐E1) to ES and the faradic by‐products from ES. It is found that pH of the culture media was not significantly changed, whereas the average hydrogen peroxide concentration was increased by 3.6 and 5.4 µM after 1 and 2 hr of ES, respectively. The upregulation of Bmp2 and Spp1 messenger RNAs was observed after 3 days of stimulation, which is consistent among two cell types. It is also found that Spp1 expression of macrophages was partially enhanced by faradic by‐products. Osteogenic differentiation of preosteoblasts was not observed during the early stage of ES as the level of Runx2 expression remains unchanged. However, cell proliferation was impaired by the excessive current density from the electrodes, and also faradic by‐products in the case of macrophages. This study shows that macrophages could respond to ES and potentially contribute to the bone formation alongside preosteoblasts. The upregulation of Bmp2 and Spp1 expressions induced by ES could be one of the mechanisms behind the electrically stimulated osteogenesis.

## Graphical abstract

In this study, the responses of macrophages and preosteoblasts to electrical stimulation (ES) were investigated. The mRNA expression from each cell type was measured after being exposed to ES. The effects of faradic by‐products were also investigated by treating the cells with electrically stimulated media. It is found in this study that Bmp2 and Spp1 mRNA expressions from both cells were upregulated by ES, whilst Spp1 mRNA expression from macrophages was partially influenced by the faradic by‐products.

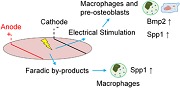

## 1 INTRODUCTION

The use of electricity in bone injury treatment was first mentioned in 1816 (Behrens, Deren, & Monchik, 2013). Until now, it has already been implemented as electrical stimulation (ES) in clinical trials and a number of devices are available commercially, which could be worth around $500 million in the US market (Haglin, Jain, Eltorai, & Daniels, 2017; Mollon, da Silva, Busse, Einhorn, & Bhandari, 2008). ES can be applied through a variety of techniques, including capacitive, inductive, direct, and combined methods (Balint, Cassidy, & Cartmell, 2013).

Capacitive ES delivers electric field through the target, whilst inductive ES delivers electromagnetic field generated by the current flowing along the solenoid (Khalifeh et al., 2018). Direct ES delivers electric field and current flow through the target alongside the faradic by‐products generated from electrochemical reactions between electrodes and the surroundings (Gan & Glazer, 2006). These ES techniques have been shown to enhance osteogenesis by promoting pro‐osteogenic protein expressions and mineralization in both in vitro (Bodamyali et al., 1998; Griffin, Sebastian, Colthurst, & Bayat, 2013; Kang et al., 2013; Mobini, Leppik, & Barker, 2016; Z. Y. Wang, Clark, & Brighton, 2006; Zhuang et al., 1997) and in vivo (P. G. Cho, Ji, Ha, Lee, & Shin, 2019; Fredericks et al., 2007; Gan, Fredericks, & Glazer, 2004; Leppik et al., 2018). However, despite these claims, the mechanism of electrically induced osteogenesis has not been fully understood yet (Khalifeh et al., 2018).

It has been shown that capacitive ES triggers calcium influx to the bone cells, and inductive ES induces calcium release from intracellular storage similar to combined ES (Brighton, Wang, Seldes, Zhang, & Pollack, 2001). These two ways of calcium movement result in an increase in osteoblast proliferation. On the contrary, electric field from direct ES causes transient increase in intracellular calcium and intracellular calcium oscillation (Hammerick, Longaker, & Prinz, 2010; Khatib, Golan, & Cho, 2004; Ozkucur, Monsees, Perike, Do, & Funk, 2009; Sun, Liu, Lipsky, & Cho, 2007), which could also promote osteogenic activities (Griffin et al., 2013; Hammerick, James, Huang, Prinz, & Longaker, 2010; Sun et al., 2007). Moreover, direct ES is capable of directing cell migration and orientation through electrotaxis and cathodic reactions (Cortese, Palama, D'Amone, & Gigli, 2014; Hammerick, James et al., 2010; Hammerick, Longaker et al., 2010; Mobini, Talts, Xue, Cassidy, & Cartmell, 2017; Tandon et al., 2009). It was also observed in vivo that new bone tissue tends to form around the cathode after applying direct ES (Baranowski, Black, Brighton, & Friedenberg, 1983; Bassett, Pawluk, & Becker, 1964; Brighton et al., 1981; Yasuda, 1977). These findings have motivated the characterization of cathodic reactions as well as their associated faradic by‐products. It is shown that cathodic ES has increased the pH, reduced O_2_ concentration, and generated reactive oxygen species (ROS) in the form of hydrogen peroxide (H_2_O_2_) (Bodamyali, Kanczler, Simon, Blake, & Stevens, 1999; Brighton, Adler, Black, Itada, & Friedenberg, 1975). These faradic by‐products are then hypothesized to be involved in the mechanisms that also support osteogenesis (Kuzyk & Schemitsch, 2009). However, it has not been tested by end‐to‐end experiment before.

The suggested mechanisms include the enhancement of osteoblastic activities at alkaline pH (Bodamyali et al., 1999; Fliefel et al., 2016; Galow et al., 2017; Ramp, Lenz, & Kaysinger, 1994), and the upregulation of vascular endothelial growth factor (Vegf) expression from macrophages by the electrically generated H_2_O_2_, which is beneficial for bone vascularization (M. Cho, Hunt, & Hussain, 2001; Griffin & Bayat, 2011). We also thought that H_2_O_2_ may also induce high‐mobility group box 1 (Hmgb1) protein from these inflammatory cells, which could potentially be beneficial for bone healing and regeneration (Meng et al., 2008; Tang et al., 2007). The involvement of macrophages in osteogenesis has been reported that they are requisite for in vivo bone healing and homeostasis, in which they have enhanced osteogenic differentiation of osteoprogenitor cells (Schlundt et al., 2015; Vi et al., 2015). Moreover, it has been shown recently that macrophages are also responsive to the electric field. Their migration, orientation, and intracellular calcium could be altered by ES similar to other cell types as well as their phagocytosis activities (Hoare, Rajnicek, McCaig, Barker, & Wilson, 2016). However, their electrically stimulated activities relating to osteogenesis are not widely studied.

Hence, it is of interest to this study to characterize the in vitro responses of macrophages to direct ES in comparison with preosteoblasts to investigate whether or not electrically stimulated macrophages could contribute to osteogenesis besides osteoblastic cells, and whether the changes in their responses are induced by faradic by‐products as hypothesized. This study provides initial proof‐of‐concept results regarding the role of faradic by‐products, which is beneficial for the future discussion regarding the mechanism of electrically induced osteogenesis.

## 2 MATERIALS AND METHODS

### 2.1 Cell culture

J774A.1 murine macrophage and phenotypically heterogeneous MC3T3‐E1 murine preosteoblastic cell lines were supplied from The European Collection of Authenticated Cell Cultures and used as received. The phenotype of J774A.1 cells used in this study were 91.4% M1 (CD11c positive), 0.6% M2 (CD206 positive), and the rest were M0 (neither CD11c nor CD206 positive) (Ono et al., 2018; Y. Zhu et al., 2017). Further details are described in the Supporting Information. Cells were maintained in Dulbecco's modified Eagle medium (4.5 g/L glucose, 2 mM l‐glutamine, without sodium pyruvate) containing 10% fetal bovine serum and 1% antibiotic and antimycotic solution. Cells were incubated at 37°C, 5% CO_2,_ and atmospheric O_2_ concentration. All reagents were purchased from Sigma‐Aldrich, UK, unless stated otherwise. In the experiments, cells were seeded into six‐well plates at the density of 50,000 cells per well for macrophages and 100,000 cells per well for preosteoblasts, and the media volume was kept at 3 ml. Media change was carried out on the following day after seeding before applying ES. Optical images of the cells were taken using EVOS™ XL Imaging System (Life Technologies).

### 2.2 Direct ES system

**Figure 1:**
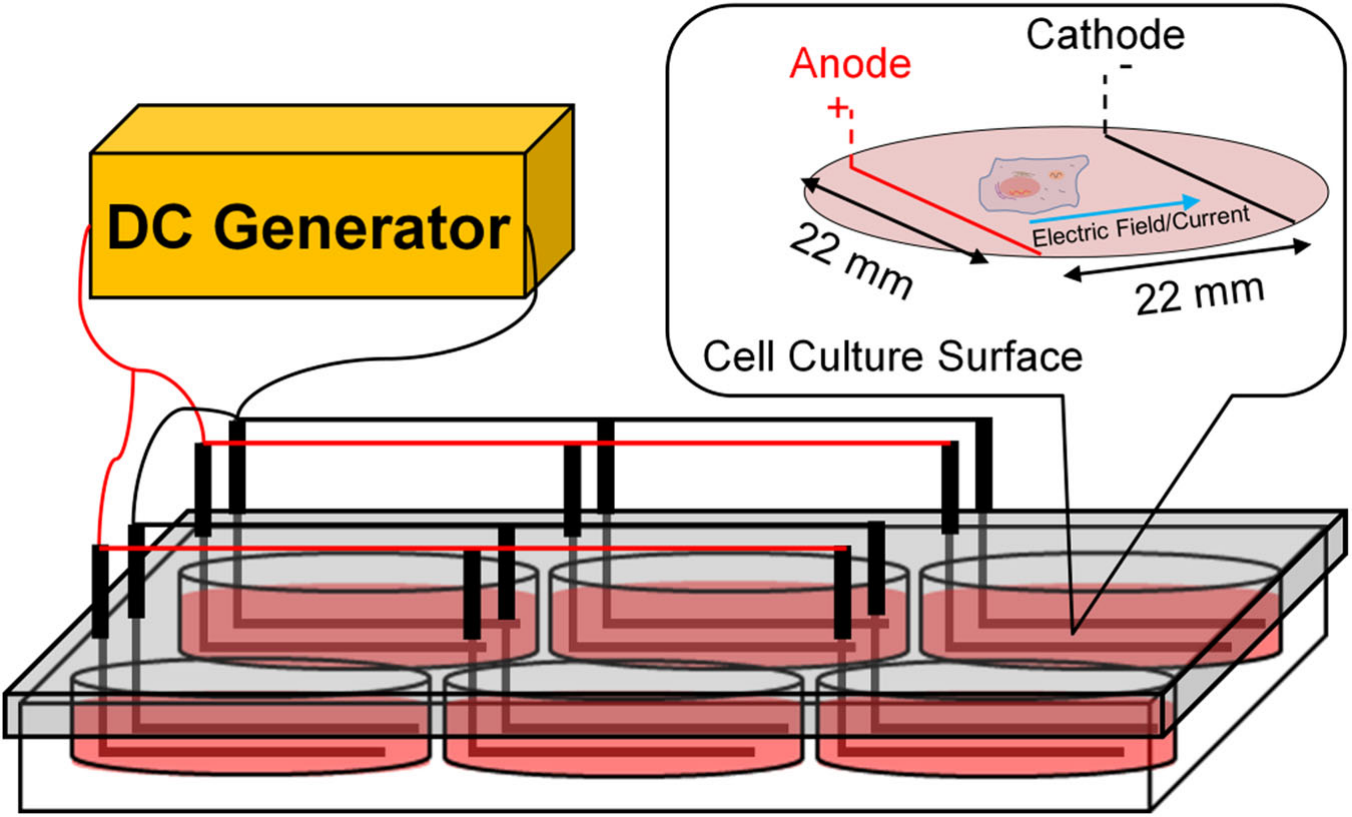
Direct electrical stimulation device with L‐shaped platinum electrodes in six‐well plate arrangement used in this study. DC, direct current. The design is adapted and modified from (Leppik et al., 2019; Mobini et al., 2016) [Color figure can be viewed at wileyonlinelibrary.com]

The system used in this study was 0.5 mm 99.95% L‐shaped platinum wire electrodes in six‐well plate arrangement (Leppik et al., 2019; Mobini et al., 2016). Electrodes were wired in parallel circuit using jumper cables, as shown in Figure 1. The devices were disinfected with 70% ethanol spray and UV irradiation before and after being used. ES has been applied to the cells (Direct ES) for 1–2 hr daily at the constant direct current (DC) voltage of 2.2 V (100 mV/mm equivalent electric field) using DC generator (B&K Precision). The total current passing through each well once reaching steady state was 0.07 ± 0.01 mA (mean ± SD) measured by digital multimeter (M‐830B, Sinometer). In faradic by‐product studies, acellular media were placed in a separate six‐well plate (3 ml per well) and incubated overnight before being stimulated for 2 hr. The electrically stimulated media (ES media) were transferred to the cells immediately after stimulation daily, and ES media were changed every time of stimulation.

### 2.3 pH measurement

Benchtop pH meter (Hanna Instruments) was used for measuring the pH of culture media. pH probe was calibrated with pH buffer between pH 4.0 and pH 10.0 (Fisher Scientific, UK) and washed with deionized water before use. Acellular media were incubated overnight before starting the experiment. The data were obtained by submerging the probe into the acellular culture media right after finishing the stimulation.

### 2.4 Fluorometric H_2_O_2_ assay

Fluorimetric Hydrogen Peroxide Assay Kit (Sigma‐Aldrich, UK) was used in this study. The reagent preparation was carried out following the supplier's instructions. Acellular media samples were incubated overnight before applying ES, and the assay was performed immediately after stimulation. The fluorescence intensity of the samples was measured after 30 min of room‐temperature incubation with the working solution at excitation wavelength of 544 nm and emission wavelength of 590 nm using microplate reader (FLUOstar® OPTIMA, BMG Labtech). The measured intensity was subtracted by background readings from nonstimulated media. H_2_O_2_ concentration was calculated using the calibration curve from standard H_2_O_2_ solution supplied with the kit.

### 2.5 Measurement of cell metabolic activity by resazurin assay

Samples were incubated with 1 ml of 10% resazurin solution (Deep Blue™ Cell Viability Kit; BioLegend, UK) diluted in the complete media for 1 hr. The fluorescence intensity of the supernatant was measured by microplate reader at excitation wavelength of 544 nm and emission wavelength of 590 nm. The background readings were subsequently subtracted from the measured intensity before analysis. The measurement was carried out the day after the final stimulation or ES media treatment.

### 2.6 Reverse transcription quantitative polymerase chain reaction (RT‐qPCR)

Samples were washed three times with Dulbecco's phosphate‐buffered saline (DPBS) and collected immediately after the final stimulation or after 2 hr of the final ES media treatment at similar exposure time as its 2‐hr direct ES counterpart. Total RNA were extracted from the samples using RNeasy Mini Kit (Qiagen, UK) following the supplier's instructions. The concentration and purity of the extracted RNA samples were measured by NanoDrop™ Lite Spectrophotometer (Thermo Fisher Scientific, UK). Complementary DNA were synthesized from 9 ng of total RNA and undergone thermal cycling following the supplier's instructions using QuantiFast SYBR® Green RT‐PCR Kit and QuantiTect Primer Assay (Qiagen, UK) alongside StepOnePlus™ Real‐Time PCR System (Applied Biosystems™, UK). Melt curves of the amplified samples were analyzed using the supplier's software. The messenger RNA (mRNA) fold expression level was calculated by comparative ΔΔC_t_ methods relative to Gapdh and control samples. The details of each primer are shown in Table 1. However, their exact sequences are proprietary.

**Table 1:** Details of primers used in this study

Genes	Assay IDs	Translated proteins
Gapdh	Mm_Gapdh_3_SG	Glyceraldehyde 3‐phosphate dehydrogenase (Gapdh)
Bmp2	Mm_Bmp2_1_SG	Bone morphogenetic protein 2 (Bmp2)
Hmgb1	Mm_Hmgb1_1_SG	High‐mobility group box 1 (Hmgb1)
Runx2	Mm_Runx2_1_SG	Runt‐related transcription factor 2 (Runx2)
Spp1	Mm_Spp1_1_SG	Osteopontin (Spp1)
Tnfα	Mm_Tnf_1_SG	Tumor necrosis factor‐α (Tnfα)
Vegfa	Mm_Vegfa_1_SG	Vascular endothelial growth factor A (Vegfa)

### 2.7 Lipopolysaccharide (LPS) treatment

J774A.1 cells were exposed to the complete media containing LPS from *Escherichia coli* O111:B4 (100 ng/ml) on the following day after seeding. Tumor necrosis factor‐α (Tnfα) mRNA expression was measured from the samples collected immediately after the first 2 hr of ES, LPS treatment, and LPS treatment with simultaneous ES as this time point was shown to exhibit the maximal level of Tnfα mRNA expression induced by LPS (Huang, Fletcher, Niu, Wang, & Yu, 2012).

### 2.8 Statistical analysis

Data were collected from the samples which were stimulated by different pairs of electrodes and statistically analyzed using GraphPad Prism 7 software. The preliminary test for normal distribution was conducted using Shapiro–Wilk normality test. The data that passed the normal distribution test were analyzed using parametric approach. The statistical test details are described in each figure caption. *p* < .05 are considered statistical significant.

## 3 RESULTS

### 3.1 pH and H_2_O_2_ measurements of acellular media

**Figure 2:**
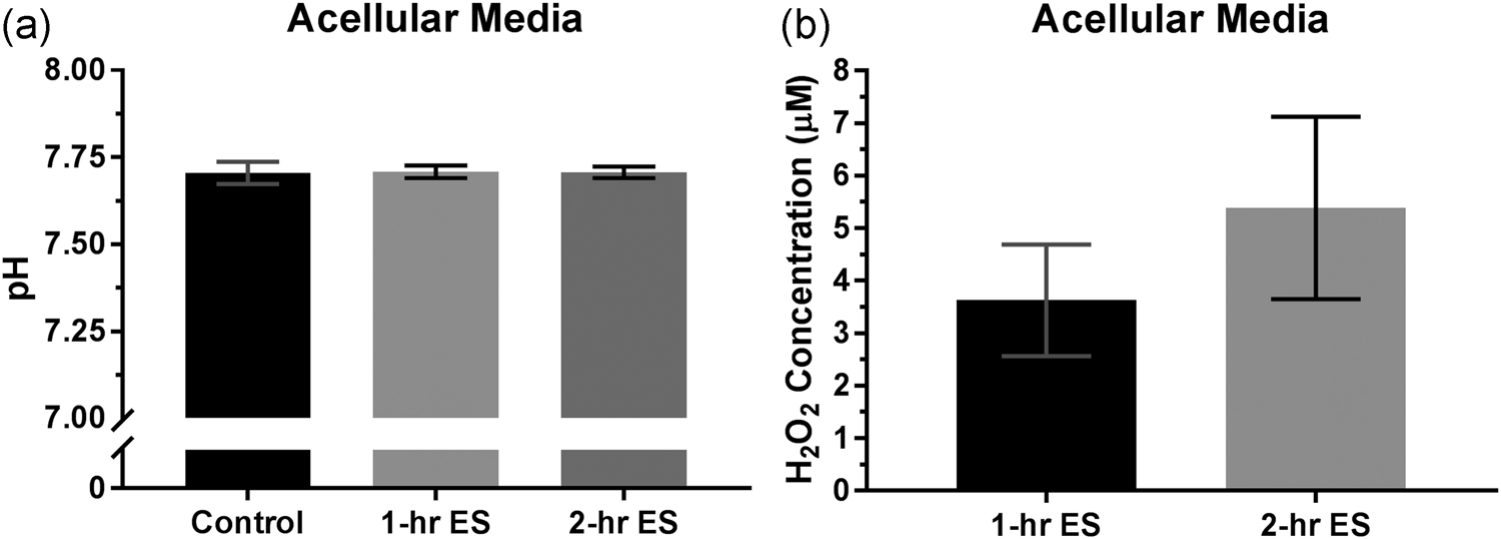
(a) pH and (b) hydrogen peroxide (H_2_O_2_) concentration of the culture media after 1 and 2 hr of electrical stimulation (ES). Error bars represent standard deviation (*n* = 6). Data were statistically analyzed by one‐way analysis of variance with Tukey's multiple comparisons test for pH and unpaired two‐tailed Student's *t* test for H_2_O_2_ concentration

Figure 2a shows the measured pH of the acellular media after 1 and 2 hr of ES. It is found that there was no significant change in pH of the media after stimulation (*p* > .96). The measured H_2_O_2_ concentration of the media after stimulation is shown in Figure 2b. The average concentration of H_2_O_2_ has increased by 3.6 and 5.4 µM after 1 and 2 hr of ES, respectively. Although the average H_2_O_2_ concentration after 2 hr of ES are around 1.5 times higher than 1 hr, the difference was not statistically significant (*p = *.06).

### 3.2 Cell metabolic activity

**Figure 3:**
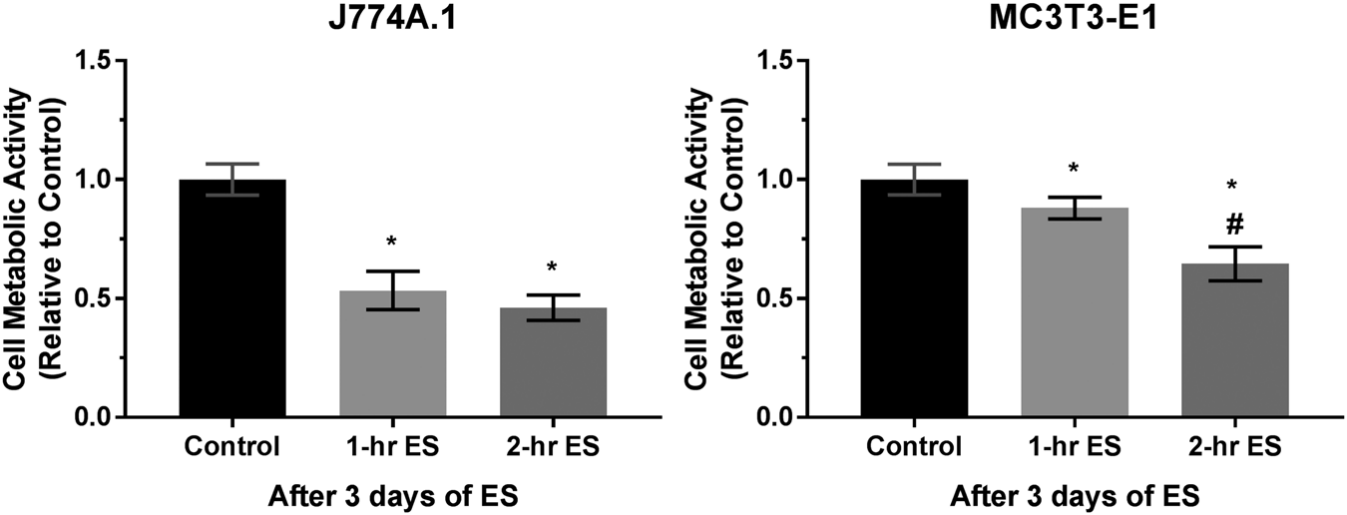
Cell metabolic activity of J774A.1 macrophages and MC3T3‐E1 preosteoblasts after 3 days of 1 and 2‐hr daily direct electrical stimulation (ES). Error bars represent standard deviation (*n* = 6). * and # represent *p* < .05 (one‐way analysis of variance with Tukey's multiple comparisons test) when compared with the control (nonstimulated) and 1‐hr daily stimulation groups, respectively

The metabolic activity of macrophages and preosteoblasts measured by resazurin assay after 3 days of stimulation are shown in Figure 3. It is found that direct ES has significantly reduced the overall metabolic activity of both cell types. However, changes in cell metabolic activity of preosteoblasts were dependent on the stimulation time, whereas those of macrophages between 1 and 2‐hr daily ES were not significantly different.

### 3.3 Cell morphology and distribution

**Figure 4:**
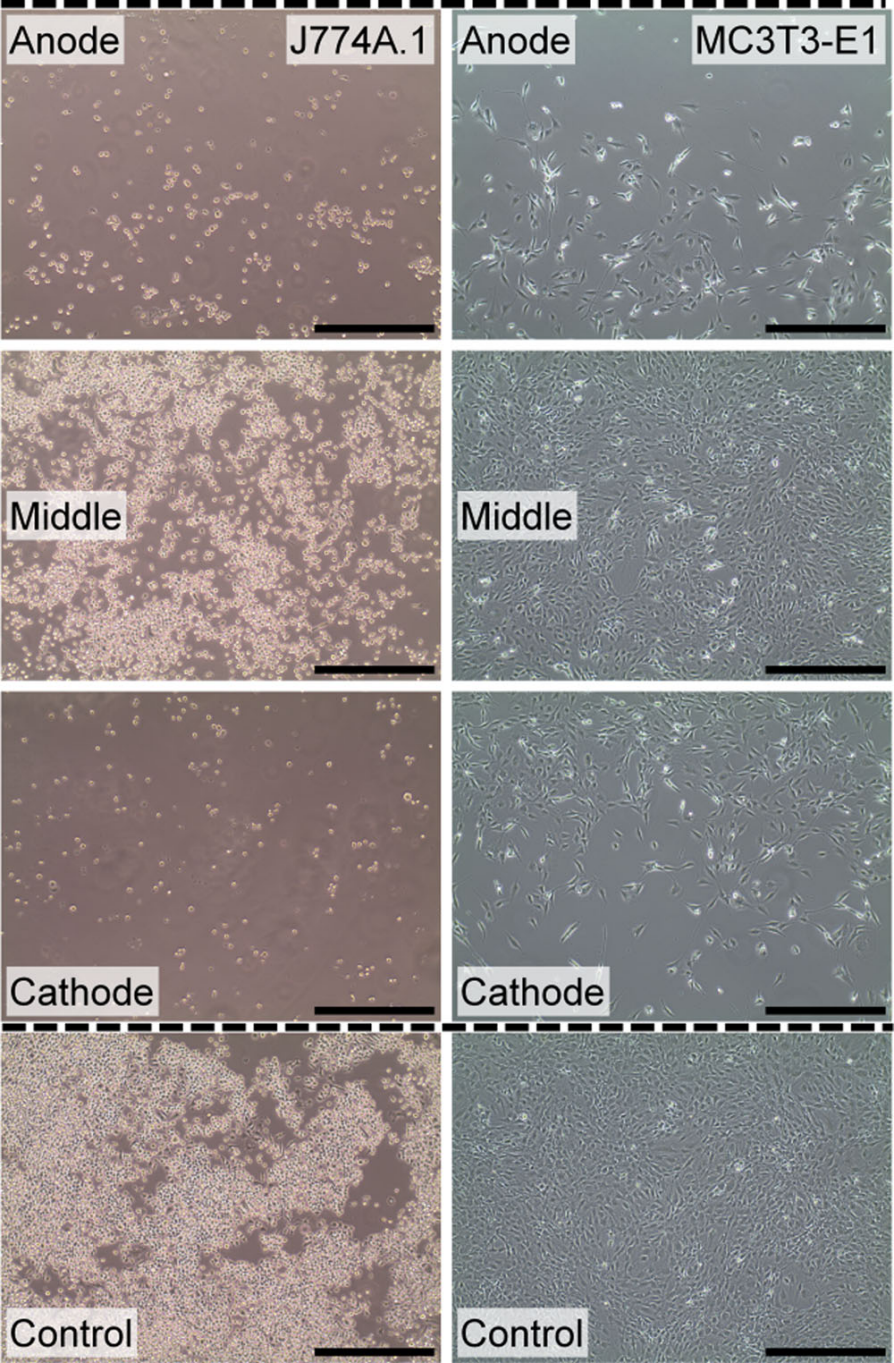
Representative optical images with ×10 objective magnification of J774A.1 macrophages and MC3T3‐E1 preosteoblasts at the area within 1,500 μm from the anodes and cathodes and in the middle of the culture area after 3 days of 2‐hr daily direct electrical stimulation. Control images represent nonstimulated cells. Scale bars = 500 μm. Dashed lines indicate electrode position [Color figure can be viewed at wileyonlinelibrary.com]

The optical images of two cell types after 3 days of 2‐hr daily ES are shown in Figure 4. It appears that the cell population were noticeably lower at the area within 1,500 μm from the anodes and cathodes than in the middle area of the well plate after 3 days of stimulation. This distribution pattern is observable in both macrophages and preosteoblasts. On the contrary, we do not observe any significant changes in cell morphology in the middle area after stimulation when compared with nonstimulated cells (control).

### 3.4 mRNA expression from RT‐qPCR

**Figure 5:**
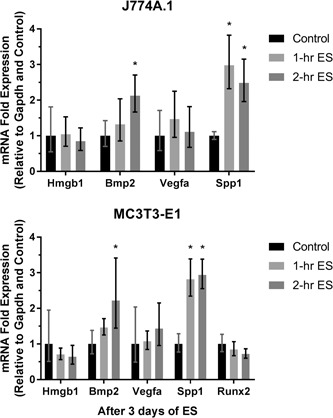
Messenger RNA (mRNA) expression of J774A.1 macrophages and MC3T3‐E1 preosteoblasts after 3 days of 1 and 2‐hr daily direct electrical stimulation (ES). Error bars represent upper and lower 95% confidence limits (*n* = 6). * represents *p* < .05 (one‐way analysis of varince with Tukey's multiple comparisons test) when compared with the control (nonstimulated) group

**Figure 6:**
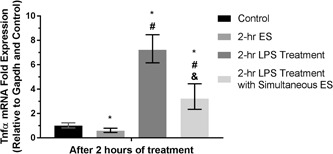
Tnfα mRNA expression of J774A.1 macrophages after 2 hr of direct ES, LPS treatment, and LPS treatment with simultaneous direct ES. Error bars represent upper and lower 95% confidence limits (*n* = 6). *, #, and & represent *p* < .05 (two‐way analysis of variance with Tukey's multiple comparisons test) when compared with the control (nonstimulated), 2‐hr ES, and LPS Treatment groups, respectively. ES, electrical stimulation; LPS, lipopolysaccharide; mRNA, messenger RNA

The expression of mRNAs from macrophages and preosteoblasts, which are responsible for translating Bmp2, Hmgb1, Vegfa, and Spp1 proteins, were evaluated in this study as well as the expression of Runx2, from preosteoblasts. It is found that Spp1 expression was upregulated after 3 days of 1‐hr daily ES in both cell types, as shown in Figure 5. Moreover, an increase in Bmp2 expression has become significant when the cells were stimulated for 2 hr daily, and the level of Spp1 expression was still comparable to those stimulated for 1 hr daily. Likewise, the upregulation of Bmp2 expression was also consistent between two cell types. However, the expression of Hmgb1 and Vegfa from these two cells were not affected by ES similarly to the Runx2 expression from preosteoblasts. Furthermore, Figure 6 shows that Tnfα mRNA expression from macrophages was reduced by ES, whereas it was increased by LPS after the first 2 hr of treatment. Besides, it is also found that the LPS‐induced Tnfα expression could be mitigated by simultaneous ES.

### 3.5 Effect of faradic by‐products

**Figure 7:**
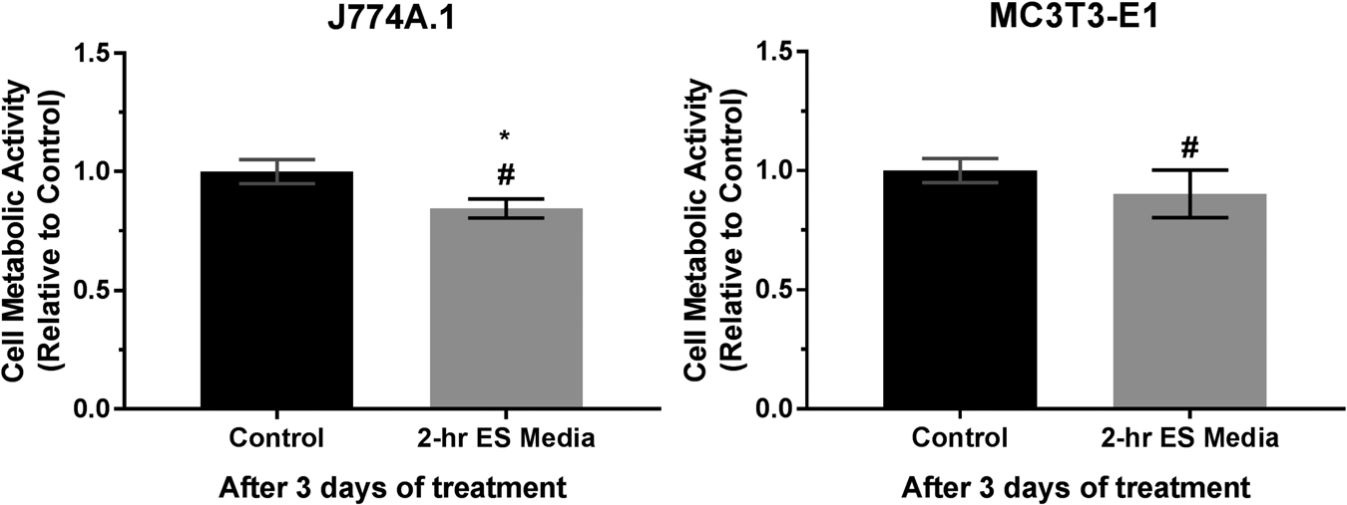
Cell metabolic activity of J774A.1 macrophages and MC3T3‐E1 preosteoblasts after 3 days of treatment with 2‐hr electrically stimulated media (ES media). Error bars represent standard deviation (*n* = 6). * and # represent *p* < .05 (unpaired two‐tailed Student's *t* test) when compared with the control (nonstimulated media) and 2‐hr daily direct ES groups, respectively

The influence of faradic by‐products as a whole was investigated by treating the cells with ES media, in which the presence of electric field, potential, and current were eliminated. The cellular responses after being exposed to the ES media in terms of the overall metabolic activity are shown in Figure 7. It can be seen that the faradic by‐products generated during the 2‐hr ES has significantly reduced the metabolic activity of macrophages after 3 days of treatment, whereas that of preosteoblasts was not significantly changed (*p* = .06). Furthermore, the metabolic activity of both cell types after ES media treatment were significantly different from direct ES group.

**Figure 8:**
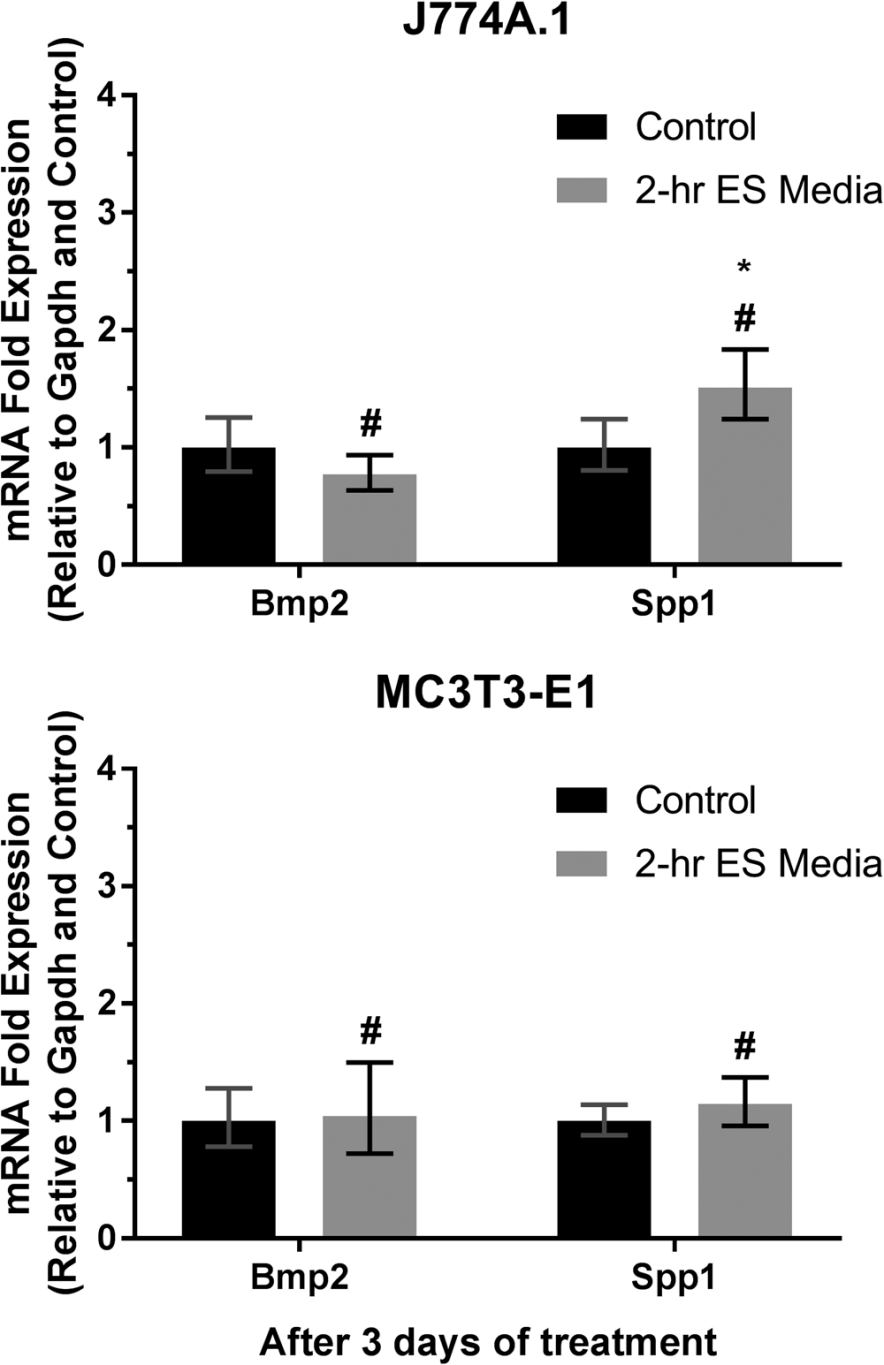
Messenger RNA (mRNA) expression of J774A.1 macrophages and MC3T3‐E1 preosteoblasts after 3 days of treatment with 2‐hr electrically stimulated media (ES media). Error bars represent upper and lower 95% confidence limits (*n* = 6). * and # represent *p* < .05 (unpaired two‐tailed Student's *t* test) when compared with the control (nonstimulated media) and 2‐hr daily direct ES groups, respectively

Changes in mRNA expressions induced by faradic by‐products are shown in Figure 8. It is seen that the mRNA expressions from two cell types were also significantly different from direct ES group. The results show that faradic by‐products have significantly upregulated the Spp1 expression of macrophages, whilst having no effect on their Bmp2 expression. Moreover, Bmp2 and Spp1 expressions from preosteoblasts were not affected by faradic by‐products.

## 4 DISCUSSION

This study has investigated the influence of direct DC ES and the faradic by‐products on the cellular responses by applying direct ES to the cells and comparing the responses with those treated with ES media. We have characterized two forms of by‐products generated during direct ES to correlate our findings with the hypothesized mechanism from the literature, which are hydroxyl ion (OH^−^) and H_2_O_2_. OH^−^ is associated with pH of the culture media, whereas H_2_O_2_ is one of the ROS that could either be beneficial for cell signaling or inducing cell oxidative stress (Bartosz, 2009). It has been studied earlier that these by‐products were the results from the radical generations through the reduction of water and oxygen during direct ES (Bodamyali et al., 1999; Kalbacova et al., 2007). Changes in extracellular environment due to these faradic by‐products could affect cellular activities in terms of proliferation and differentiation (Galow et al., 2017; Li et al., 2009). It was shown previously that cathodic stimulation with titanium electrodes has reduced the metabolic activity of osteoblasts and macrophages and increased their intracellular ROS level (Kalbacova et al., 2007). In this study, we have further discussed this finding in terms of their pro‐osteogenic mRNA expressions and also demonstrated that the effects of faradic by‐products on cellular activities are not dominant compared with the effects of ES. Moreover, we also found that macrophages respond to ES in a similar manner as preosteoblasts, and it is not only faradic by‐products that stimulate macrophage activities during electrically induced osteogenesis.

### 4.1 Reduction in cell viability after stimulation

It was shown that pH changes after direct ES is dependent on the stimulation regime and excessive increase in pH may lead to the reduction in cell viability (Balint, Cassidy, Hidalgo‐Bastida, & Cartmell, 2013). Although our results show that changes in pH are not significant during stimulation, the reduction in overall cell metabolic activity indicates that the population of metabolically active cells, which are the viable cells, is still reduced after being stimulated. This could be due to the other type of faradic by‐products. However, the optical images at different areas of the stimulated cells have shown that the apparent cell density at the vicinity of the electrodes is noticeably lower than those in the middle of the well. Besides, platinum electrodes have already been tested for its biocompatibility (Geninatti et al., 2015). Therefore, we believe that the excessive current density around the electrodes during stimulation has significant effects on cell viability and proliferation in our ES system (Balint, Cassidy, Hidalgo‐Bastida et al., 2013).

On the contrary, cell metabolic activity after being exposed to ES media are significantly higher than those stimulated by direct ES. This confirms the negative effects of excessive current density on cell proliferation when the electrodes are present during direct ES. Moreover, it is also found that faradic by‐products could also reduce macrophage proliferation. We initially thought that H_2_O_2_ might be the reason behind this; however, it has been reported that cell oxidative stress would occur at H_2_O_2_ concentration above 10 μM, which is higher than those generated from our ES system (Stone & Yang, 2006; X. Y. Wang et al., 2015). Moreover, the preosteoblastic and macrophage cell lines are capable of tolerating more than 100 μM of exogenous H_2_O_2_ (Arai, Shibata, Pugdee, Abiko, & Ogata, 2007; Tang et al., 2007). Hence, it is possible that the reduction in macrophage proliferation could be due to other type of faradic by‐products, which have not been characterized yet.

### 4.2 Potential electrically stimulated osteogenesis mechanism

The RT‐qPCR results have shown the similar changes in mRNA expression pattern between macrophages and preosteoblast as a result of ES. Spp1 expression has increased with 1‐hr daily stimulation, whereas the expression of Bmp2 requires 2 hr of stimulation daily. It is understood that 2 hr daily are the optimal stimulation time for this system as it could induce the upregulation of both Bmp2 and Spp1.

It has been reported that Bmp2 is essential for the early stage of bone healing, which subsequently enhances differentiation of osteoprogenitor cells and their migration towards the injury site as well as bone formation (Knippenberg, Helder, Doulabi, Wuisman, & Klein‐Nulend, 2006; Rickard, Sullivan, Shenker, Leboy, & Kazhdan, 1994; Tsuji et al., 2006; Wozney, 1992). Likewise, Spp1 was also found in the early stage of bone healing reportedly secreted by macrophages (McKee & Nanci, 1996; McKee, Pedraza, & Kaartinen, 2011). This Spp1 could also support the osteogenic differentiation of osteoprogenitor cells (Chen et al., 2014). However, the upregulation of Vegfa and Hmgb1 from macrophages were not observed, although it is suggested earlier in the literature as well as being hypothesized in this study. This could be due to the large difference between the H_2_O_2_ concentration used in the literature (>100 μM) and those generated from ES (<10 μM) (M. Cho et al., 2001; Tang et al., 2007). The expression of Vegf from preosteoblasts may also be dependent on ES regime. It has been shown that MC3T3‐E1 preosteoblastic cells expressed higher level of Vegf after being stimulated by biphasic pulses, which is not observed when stimulated with constant DC in this study (Kim et al., 2006).

Furthermore, the level of Runx2 expression was not significantly changed after 3 days of stimulation, which indicates that there was no change in cellular activities related to the maturation of preosteoblasts during this period (Komori, 2010). This suggests that direct ES may not promote osteogenesis by inducing cellular osteogenic differentiation or osteoblast maturation directly during the early stage, but rather promote pro‐osteogenic mRNA expressions from macrophages and preosteoblasts. In the case that these differential mRNA changes have been translated into their associated proteins, these proteins would then enhance bone regeneration from the native tissues or cells. It is expected that the upregulation of Bmp2 and Spp1 expressions would be one of the mechanisms behind the electrically stimulated osteogenesis.

The upregulation of Bmp2 expression after direct ES is also consistent with previous in vivo study. It was reported that the fractured bone tissue expresses Bmp2 after applying direct ES, and the expression of Vegf was not increased (Fredericks et al., 2007). Furthermore, Bmp2 expression could also be enhanced by capacitive ES technique in both in vitro and in vivo (Gan et al., 2004; Griffin et al., 2013; Z. Y. Wang et al., 2006) as well as inductive ES in vitro (Bodamyali et al., 1998). It was previously thought that Bmp2 was produced by osteoblasts as a result of in vivo ES (Griffin & Bayat, 2011). However, it is found that Bmp2 and Spp1 are also expressed from electrically stimulated mesenchymal stem cells as well as macrophages observed in this study (Leppik et al., 2018; Mobini et al., 2016; Mobini, Leppik, Parameswaran, & Barker, 2017).

Interestingly, this study shows that Bmp2 and Spp1 mRNA expressions are primarily a result of ES, whereas faradic by‐products have partially contributed to an increase in Spp1 expression from macrophages, potentially involving 4EPB‐1 translation or nuclear factor‐κB and AP‐1 transcription (Lyle et al., 2014). Apart from pH and H_2_O_2_, faradic by‐products also include H_2_ generation, O_2_ reduction, and any other surrounding molecules generated by redox reactions during ES (Bodamyali et al., 1999; Brighton et al., 1975; Brummer, Mchardy, & Turner, 1977; Merrill, Bikson, & Jefferys, 2005). These changes could affect cellular activities, and they have not been fully characterized yet. Therefore, we are unable to confirm whether it was H_2_O_2_ alone, or other faradic by‐products, or both, which triggered this response based on our current results. Despite the discussion that faradic by‐products could possibly be behind the upregulation of Bmp2 mRNA expression after ES, we clearly demonstrate that the increased Bmp2 mRNA expression is more likely a result of ES than the by‐products (Fredericks et al., 2007; Gan & Glazer, 2006). It would also be worth investigating further in details regarding how ES interacted with cells, and whether or not it is the interaction with voltage‐sensitive ion channels as discussed in the literature that triggers these mRNA expressions from macrophages and preosteoblasts (Balint et al., 2013; Thrivikraman, Boda, & Basu, 2018).

### 4.3 Responses of macrophages to direct ES

Macrophages are well known for triggering inflammatory responses; however, they could also be beneficial for wound healing and tissue regeneration depending on their phenotypes. The proinflammatory phenotype is M1 macrophages and wound‐healing phenotype is M2, and the differentiation towards these two phenotypes is dependent on their microenvironment and stimuli (Murray, 2017; Rőszer, 2015; Y. Zhang et al., 2013). It is understandable that the increased Spp1 mRNA expression from preosteoblasts is the marker for their activity along the osteoblastic lineage (Rutkovskiy, Stensløkken, & Vaage, 2016). However, Spp1 is also involved in the function, migration, and differentiation of macrophages (Bruemmer et al., 2003; Lund, Giachelli, & Scatena, 2009; Nyström, Dunér, & Hultgårdh‐Nilsson, 2007). Hence, the upregulation of Spp1 expression from macrophages could have diverse interpretation (Lund et al., 2009). Apart from its roles in facilitating soft tissue and bone remodeling, Spp1 expression could also be a sign of macrophage maturation and inflammation, which can lead to the adverse effects on osteogenesis (Gilbert et al., 2000; Krause et al., 1996; Liaw et al., 1998; Lund et al., 2009; Ogawa et al., 2005; Rittling et al., 1998; Saleh, Carles‐Carner, & Bryant, 2018; Zhao et al., 2011).

It is found from this study that direct ES reduced Tnfα mRNA expression from macrophages, which is a proinflammatory and anti‐M2 macrophage marker that could be triggered by LPS (Huang et al., 2012; Murray, 2017). In addition, it has also been shown previously that the Bmp2 mRNA expression from J774A.1 cells was not from proinflammatory activities (Champagne, Takebe, Offenbacher, & Cooper, 2002). Therefore, it is plausible that the increased Spp1 and Bmp2 mRNA expressions after direct ES were not indicative of the proinflammatory responses. Nonetheless, it could still be asked whether the status of macrophages remained unchanged or polarized towards M2 as the recent study has suggested that the increased Spp1 mRNA expression is also a characteristic of the M2c subtype of M2 macrophages, including J774A.1 cell lines (Capote et al., 2016).

In addition to the findings from this study, the recent in vivo study also reports that ES has significantly reduced the inflammation during bone injury, which is consistent with the conclusion from an analysis of clinical studies suggesting that ES is effective in reducing pain and radiographic nonunion‐healing rate (Aleem et al., 2016; Fonseca et al., 2018). Moreover, the faradic by‐products generated during direct ES also have potential antibacterial effects which could be capable of reducing the risk of infection (Asadi & Torkaman, 2014). Hence, it would be worth investigating further on the anti‐inflammatory effects of ES and to determine whether or not ES could induce macrophage polarization towards any proregenerative M2 subtypes in future.

### 4.4 Further optimization of direct ES system and the limitations of this study

It is seen from this study that although the majority of cellular responses are induced by ES, the faradic by‐products also have an effect on cells. Therefore, further characterization of the ES media may be requisite to explore the unidentified faradic by‐products and their effects on cellular activities, such as the radicals present during the process of H_2_O_2_ generation (Kalbacova et al., 2007). The insulation of the electrodes could be implemented in future to deconstruct the effects of direct ES and faradic by‐product generation on cellular activities. At the same time, the current density near the electrodes needs to be minimized. It was suggested that the optimal current density for promoting osteogenesis using platinum electrodes is between 1 and 5 A/m^2^, and exceeding this range would result in tissue or cell necrosis (Spadaro & Becker, 1979). Otherwise, novel techniques could be developed and implemented to prevent cell penetration into the invasive region, such as surface modification. The excessive electrolysis of culture media should also be avoided during the stimulation as it can cause adverse effects and cytotoxic complications (Thrivikraman et al., 2018). Moreover, it is worth investigating the electrically stimulated cells further, in terms of their protein secretion and interactions with other osteoprogenitor cells, as well as the variation in cellular activities between tumor‐associated macrophage cell lines used in this study and primary bone marrow macrophages present at bone injury sites (Chamberlain, Godek, Gonzalez‐Juarrero, & Grainger, 2009; Murray, 2017; Saleh et al., 2018). The incorporation of this direct ES system with conductive scaffolds could also be a promising approach as the recent studies show that it could have significant improvement in promoting cell proliferation and/or osteogenic activities (Hu, Chen, Tsao, & Cheng, 2019; J. Y. Zhang, Li, Kang, & Neoh, 2016; S. Zhu et al., 2017).

## 5 CONCLUSION

ES could promote bone regeneration by inducing Bmp2 and Spp1 mRNA expressions from macrophages and preosteoblasts. Preosteoblasts did not respond to faradic by‐products in terms of Bmp2 and Spp1 mRNA expressions, whereas macrophages responded by increasing their Spp1 expression. We demonstrated that the roles of faradic by‐products are not dominant, and the by‐products alone would be less effective in promoting bone formation without the presence of ES. The findings from this study also imply that cellular responses from preosteoblasts and macrophages to ES are predominantly triggered by the mechanism involving electric field, potential, and/or current. On the contrary, Vegfa and Hmgb1 mRNA expressions from both types of cells and Runx2 expression from preosteoblasts are not affected by the ES regime used in this study. In addition, we showed that this ES system may need further optimization to reduce the current density near the electrodes, which locally impairs cell viability and proliferation.

## CONFLICT OF INTERESTS

The authors declare that there are no conflict of interests.

## Supporting information

Supplementary information
