# Supplementary material for: Direct electrical stimulation enhances osteogenesis by inducing Bmp2 and Spp1 expressions from macrophages and preosteoblasts
Source: Biotechnol Bioeng. 2019 Sep 23;116(12):3421–32. doi: 10.1002/bit.27142 (PMC6899728; doi:10.1002/bit.27142)
Supplement: Supplementary file 1 — Supplementary information [file BIT-116-3421-s001.docx]

**Supplementary Information**

Supplementary Materials and Methods

*The phenotypic characterisation of J774A.1 cell stock*

CD11c and CD206 were used as markers for M1 and M2 phenotype, respectively (Ono et al., 2018; Zhu et al., 2017). Conjugated Alexa Fluor^®^ 594 anti-mouse CD11c and Alexa Fluor^®^ 488 anti-mouse CD206 (MMR) antibodies were purchased from BioLegend. Other reagents were purchased from Sigma-Aldrich, unless stated otherwise. 20,000 cells were seeded onto the rectangular glass coverslip (12 x 12 mm^2^) in 12-well plate. On the following day after seeding, cells were washed with DPBS and fixed for 10 minutes with 10% neutral buffered formalin solution at room temperature. Subsequently, cells were washed again with DPBS before and after 10-minute permeabilisation with 0.2% Triton^®^ X-100 in DPBS at room temperature. The permeabilised cells were blocked with 10% goat serum in DPBS containing 1% bovine serum albumin (BSA) and 0.1% Tween^®^ 20 (VWR) at room temperature for 30 minutes. Two antibodies were diluted 100 times in DPBS containing 1% BSA and 0.1% Tween^®^ 20 and incubated with the cells at 4°C overnight. Cells were subsequently washed with DPBS containing 1% BSA and 0.1% Tween^®^ 20 and stained with 5 µg/ml 4’, 6-diamidino-2-phenylindole (DAPI) (Invitrogen) at room temperature for 1 hour. The stained cells were washed again with DPBS containing 1% BSA and 0.1% Tween^®^ 20 and mounted on the glass microscope slides using Fluoroshield Mounting Medium (Abcam). Fluorescence images were taken by confocal microscopy (Leica, SP8). The stained cells were quantified from 5 images taken from 3 coverslips by CellProfiler^™^ software version 3.0.0 with the total cell number of 631 cells.

Supplementary Figure

**
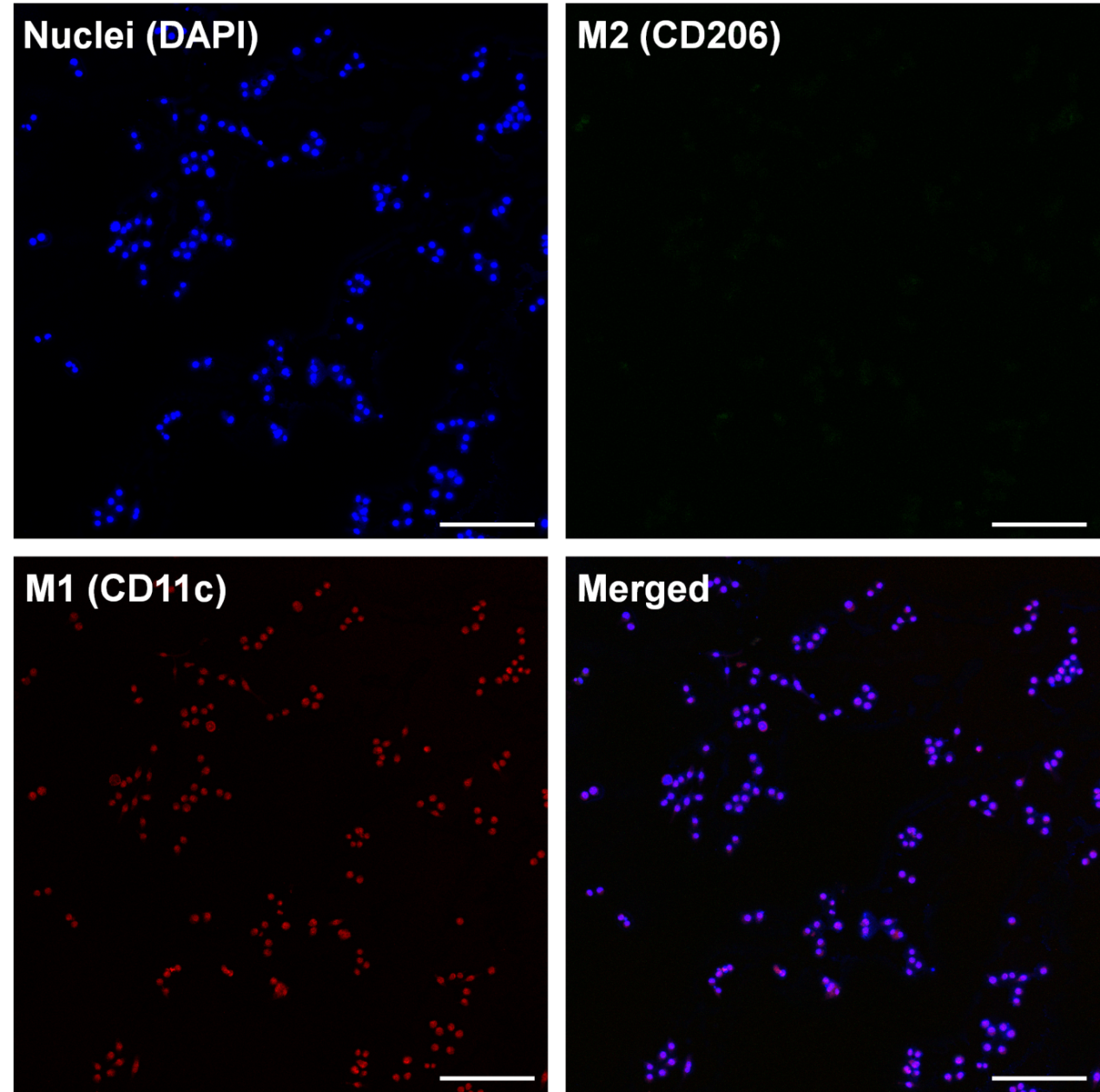
**

**Supplementary Figure S1** Representative fluorescence images of J774A.1 cells used in this study. 10X objective magnification. Scale bar = 200 µm.

**References**

Ono, Y., Nagai, M., Yoshino, O., Koga, K., Nawaz, A., Hatta, H., . . . Saito, S. (2018). CD11c+ M1-like macrophages (MΦs) but not CD206+ M2-like MΦ are involved in folliculogenesis in mice ovary. *Scientific Reports, 8*(1), 8171. doi:10.1038/s41598-018-25837-3

Zhu, Y., Zhang, L., Lu, Q., Gao, Y., Cai, Y., Sui, A., . . . Xie, B. (2017). Identification of different macrophage subpopulations with distinct activities in a mouse model of oxygen-induced retinopathy. *International Journal of Molecular Medicine, 40*(2), 281-292. doi:10.3892/ijmm.2017.3022
